# Green Synthesis of Gold Nanoparticles Using *Polianthes tuberosa* L. Floral Extract

**DOI:** 10.3390/plants10112370

**Published:** 2021-11-03

**Authors:** Mousa A. Alghuthaymi, Chandrasekaran Rajkuberan, Thiyagaraj Santhiya, Ondrej Krejcar, Kamil Kuča, Rajiv Periakaruppan, Seetharaman Prabukumar

**Affiliations:** 1 Biology Department, Science and Humanities College, Shaqra University, Alquwayiyah 19245, Saudi Arabia; malghuthaymi@su.edu.sa; 2 Department of Biotechnology, Karpagam Academy of Higher Education, Coimbatore 641021, India; santhiathiyagaraj1964@gmail.com (T.S.); rajivsmart15@gmail.com (R.P.); 3 Malaysia Japan International Institute of Technology (MJIIT), Universiti Teknologi Malaysia, Kuala Lumpur 54100, Malaysia; ondrej.krejcar@uhk.cz; 4 Department of Chemistry, Faculty of Science, University of Hradec Kralove, 50003 Hradec Kralove, Czech Republic; kamil.kuca@uhk.cz; 5 Department of Biotechnology, Bharathidasan University, Tiruchirappalli 620024, India; prabukmr89@gmail.com; 6 Laboratory of Functional Molecules and Materials, School of Physics and Optoelectronic Engineering, Shandong University of Technology, Zibo 255000, China

**Keywords:** biosynthesis, *Polianthes tuberosa*, flower, gold, nanoparticles, *E. coli*, cytotoxicity

## Abstract

The developments of green-based metallic nanoparticles (gold) are gaining tremendous interest, having potential applications in health care and diagnosis. Therefore, in the present study, *Polianthes tuberosa* flower filtered extract was used as a reducing and stabilizing agent to synthesize gold nanoparticles (PtubAuNPs). The PtubAuNPs were extensively characterized by UV–visible spectroscopy, Fourier transform infrared spectroscopy, transmission electron microscopy, and X-ray diffraction. The antibacterial activity of PtubAuNPs was determined by the agar well diffusion method; the PtubAuNPs performed extreme antagonistic activity against the tested pathogens. Furthermore, the cytotoxicity of the PtubAuNPs was evaluated in MCF 7 cells by MTT assay. The PtubAuNPs induced toxicity in MCF 7 cells with the least concentration of 100 µg/mL in a dose-dependent method by inducing apoptosis. Overall, the study manifested that PtubAuNPs are a potent nanomaterial that can be employed as an antimicrobial and anticancer agent.

## 1. Introduction

Customized nanoparticles in the size range of 1–100 nm are gaining stupendous attraction from researchers globally. This is because nanoparticles are potent materials having unique optical, physical, chemical, and conductive properties [1]. Moreover, these materials have been widely employed in various chemical, physical, and biological science fields [2].

Nanoparticles possess a uniquely large surface-to-volume ratio and exhibit various size forms that have unique physical and chemical characteristics compared with bulk materials [3]. Accordingly, based on the structure of the nanoparticles; the functional properties will vary. The transition from bulk materials to micro/nano changes the properties. This is due to the interatomic bonding present in the large crystals, which is not found on an atomic level surface, and henceforth the atoms become more reactive [4]. Such materials markedly show superior properties and possess numerous applications.

Metallic nanoparticles such as silver (Ag), gold (Au), and iron (Fe) show numerous beneficial properties, and are applied in all industrial sectors. Noticeably, gold nanoparticles are the choice of interest for researchers compared to other metals, owing to their stupendous optical properties. Moreover, gold nanoparticles can be fabricated through various synthetic methods (physical, chemical) to produce nanoparticles with variant sizes and shapes, including nanospheres, nanorods, nanocubes, nanobranches, nanobipyramids, nanoflowers, nanoshells, nanowires, and nanocages [5]. In the past few years, the green chemistry perspective has become a significant method for the synthesis of gold nanoparticles via a bottom-up approach [6]. Through this approach, plants (leaves, flowers, fruits, seeds, stems, roots, and latex) and microbial extracts (bacterium, fungi, and actinomycetes) were employed as a reducing/stabilizing agent for the synthesis of gold nanoparticles [7]. The above method is clean, nontoxic, avoids the use of hazardous chemicals and reagents, and is amenable to biological applications [8].

Gold nanoparticles have myriad applications in biomedical fields; this is due to their properties such as biocompatibility, surface chemistry, high binding affinity, enhanced solubility, and tunable functionalities for targeted drug delivery [9]. Moreover, gold nanoparticles can be easily conjugated with drugs, polymers, proteins, enzymes, DNA/RNA, and other chemical moieties to enhance the biological activity in various infectious/noninfectious diseases [10]. In addition, gold nanoparticles are a photothermal material that converts light energy into heat energy, and are widely used in cancer treatment [11]. Gold nanoparticles have excellent diagnostic and imaging attributes due to the phenomena of localized surface plasmon resonance (LSPR) and surface-enhanced Raman scattering (SERS) [12].

Generically, flowers have an aesthetic appearance, which activates the visual region of the brain, viscera motor, sensory motor, and cerebral circuits [13]. Commercially, flowers have a global market trade for use as a whole, and in oils and perfumes. Medically, flowers possess therapeutic properties; for instance, *Hibiscus rosasinensis* flower, *Mimusops elengi* flower, *Nyctanthes arbor-tristis* flower, and *Tussilago farfara* flower bud were reported to have various therapeutic activities [14]. Polianthes tuberosa flower is predominantly used in the perfume industry due to the presence of aromatic compounds. In addition, the plant contains various metabolites such as flavonoids and steroid glycosides, and the flower contains benzoid and terpenoid dervatives as the major metabolites [15].

Using the above rationale, we constructed this study to synthesize gold nanoparticles from floral aqueous extract of *Polianthes tuberosa* L. The synthesized gold nanoparticles were fabricated through a benign approach and characterized through UV–visible spectroscopy, Fourier transform infrared spectroscopy, transmission electron microscopy, and X-ray diffraction. Moreover, the gold nanoparticles were assessed for their antibacterial and anticancer activities.

## 2. Materials and Methods

### 2.1. Floral Extract Preparation

**Figure 1:**
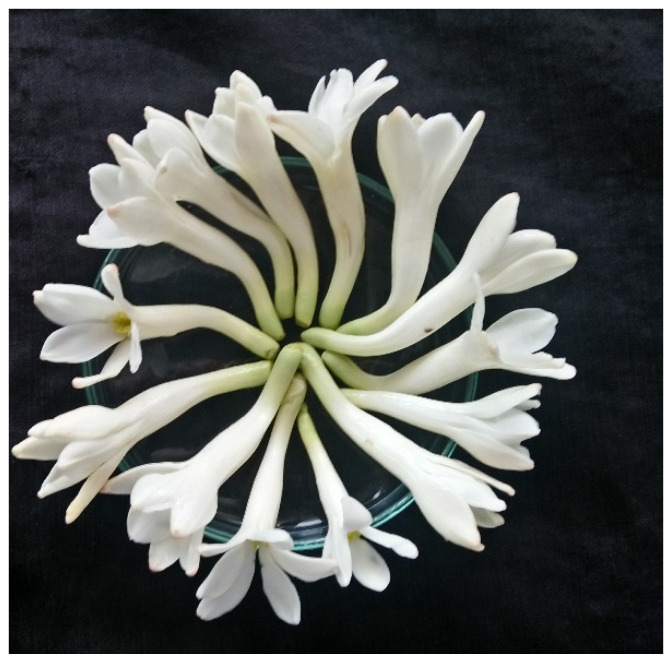
An image of a *P. tuberosa* flower.

*P. tuberosa* flowers were purchased from the local market and brought immediately to the lab, then the flowers were washed with distilled water and sliced into smaller pieces and kept air-dried at room temperature for one week (Figure 1). After that week, the dried flowers were blended into a fine powder and stored in an airtight container until further use. For floral aqueous extract preparation; 50 g of powder was added to 250 mL of boiling distilled water and kept on a magnetic stirrer plate at 60 °C and 100 RPM for 30 min. After the time interval, the suspension was filtered through Whatman grade 1 filter paper, and the filtered extract was stored at 4 °C for further use.

### 2.2. Synthesis of P. tuberosa Floral Gold Nanoparticles (PtubAuNPs)

To synthesize the PtubAuNPs, the floral extract was mixed in the gold chloride solution in the proportion of stoichiometric ratio and kept in a heating mantle at the requisite temperature and time with the desired pH and observed visually for color changes. Initially, we applied various optimization parameters for the floral extract (1–10 mL): chloroauric acid (HAuCl_4_) (1–2.5 mM); temperature (40–80 °C); pH (4, 7, 9); time (0–300 min) and stoichiometric proportion. Finally, for fine generation of PtubAuNPs, the following reaction kinetics were fixed: floral extract 3 mL; HAuCl_4_ 1.5 mM; temperature 60 °C; pH 9; time 60 min; and stoichiometric proportion (7:3).

### 2.3. Characterization of P. tuberosa Floral Gold Nanoparticles (PtubAuNPs)

The synthesized PtubAuNPs were first assessed using UV–vis spectroscopy (Shimadzu UV-1601 dual-beam spectrophotometer, Tokyo, Japan) operated at a resolution of 1 nm in a wavelength of range 200–800 nm, and distilled water was used as a blank. For Fourier transform infrared spectroscopy (FTIR), a Perkin Elmer RX1 was used to detect the presence of functional groups present in the extract and synthesized PtubAuNPs. The analysis was carried out on a Perkin-Elmer Spectrum FTIR system. The spectrum was obtained in transmittance mode operated at a resolution of 2 cm^−1^ from 4500 cm^−1^ to 500 cm^−1^. The morphological features of the PtubAuNPs were ascertained by transmission electron microscopy (TEM) analysis. Before analysis, the powder sample (PtubAuNPs) was dispersed in aqueous solution and was coated on copper grids and left to dry. The analysis was performed with a JOEL 3010 instrument, and the elemental composition was determined by energy dispersive spectroscopy (EDX) coupled with the instrument. The crystalline nature and orientation of the PtubAuNPs were authenticated using X-ray diffraction (XRD) analysis using an Ultima IV X-ray powder instrument with CuKα radiation (Rigaku Ltd., Tokyo, Japan).

### 2.4. Antibacterial Evaluation of PtubAuNPs

For analysis, MTCC cultures were procured from the Institute of Microbial Technology, Chandigarh. The chemicals Mueller–Hinton agar and gentamicin were purchased from Sigma Aldrich, India. The antibacterial activity of the synthesized PtubAuNPs was evaluated against the Gram negative *Escherichia coli* (MTCC no.: 443) and Gram-positive *Staphylococcus aureus subsp. aureus* (MTCC no.: 737) using the agar well diffusion method [16]. Briefly, the bacterium was cultured in the respective medium for overnight incubation at room temperature. Freshly prepared Mueller–Hinton agar (MHA) plates with a well of diameter 6 mm were made. Using a sterile cotton swab, the cultures were uniformly streaked around the plate, and the test sample (PtubAuNPs) was loaded into the well at different concentrations (20, 40, or 60 µg/mL) and kept in the incubator at 37 °C overnight. The standard drug gentamicin was used as a positive control (10 µg/mL). After the incubation period, the zone of inhibition was measured and expressed in mm.

### 2.5. Cytotoxic Assessment of PtubAuNPs

The MCF 7 breast cancer cell line was employed to address the cytotoxic potential of the PtubAuNPs [17]. The MCF 7 cells were procured from the National Centre for Cell Science (NCCS) Pune, and grown in Eagle’s Minimum Essential Medium with 10% fetal bovine serum (FBS) and maintained at 37 °C, 5% CO_2_, 95% air, and 100% relative humidity. The cells were routinely passaged weekly, and the culture medium was changed twice a week.

Before the assay, the monolayer cells were subjected to trypsin/EDTA treatment to form single-cell suspensions; the viable cells were counted using a hemocytometer and diluted with a medium containing 5% FBS to reach a final density of 1 × 10^5^ cells/mL. In 96-well plates, 100 µL of cell suspensions was added into the well at a plating density rate of 10,000 cells/well and incubated to allow for cell attachment at 37 °C, 5% CO_2_, 95% air, and 100% relative humidity. After 24 h, the cells were treated with serial concentrations of the test samples. They were initially dispersed in phosphate-buffered saline (PBS) and diluted to twice the desired final maximum test concentration with serum-free medium. An additional four 2-fold serial dilutions were made to provide a total of five sample concentrations. Aliquots of 100 µL of these different sample dilutions were added to the appropriate wells already containing 100 µL of medium, resulting in the required final sample concentrations. The medium without samples served as control, and the triplicate assay was performed.

Next, 15 µL of 3-[4,5-dimethylthiazol-2-yl]2,5-diphenyltetrazolium bromide (MTT) (5 mg/mL) in phosphate-buffered saline (PBS) was added to each well and incubated at 37 °C for 4 h. The medium with MTT was then turned off, and the formed formazan crystals were solubilized in 100 µL of DMSO, and then we measured the absorbance (Abs) at 570 nm using a microplate reader [16].

The % cell inhibition was determined using the following formula:

% Cell Inhibition = 100 − Abs (sample)/Abs (control) × 100.

A nonlinear regression graph was plotted between % cell inhibition and log concentration, and IC50 was determined using GraphPad Prism software.

## 3. Results and Discussion

The exploitation of natural resources in nanotechnology is needed in the current epoch to develop smart materials for humankind and the environment. In the past few decades, myriad reports were published in the context of metallic nanoparticles synthesized using plants [18]. In continuation of this, the synthesis of gold nanoparticles using flower extracts is also garnering significant attention in the scientific community. In the present study, we have provided the updated status of flower mediated synthesis of gold nanoparticles as Table 1.

**Figure 2:**
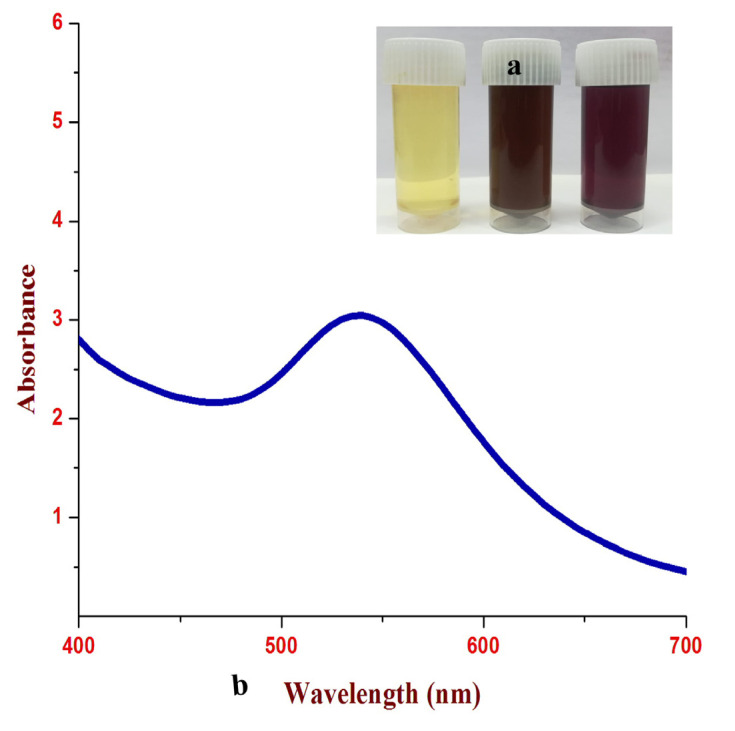
(**a**) Synthesis of PtubAuNPs (from right to left): gold chloride solution, *Polianthes tuberosa* flower extract, and gold nanoparticles; (**b**) the UV–vis spectrum of the PtubAuNPs.

**Figure 3:**
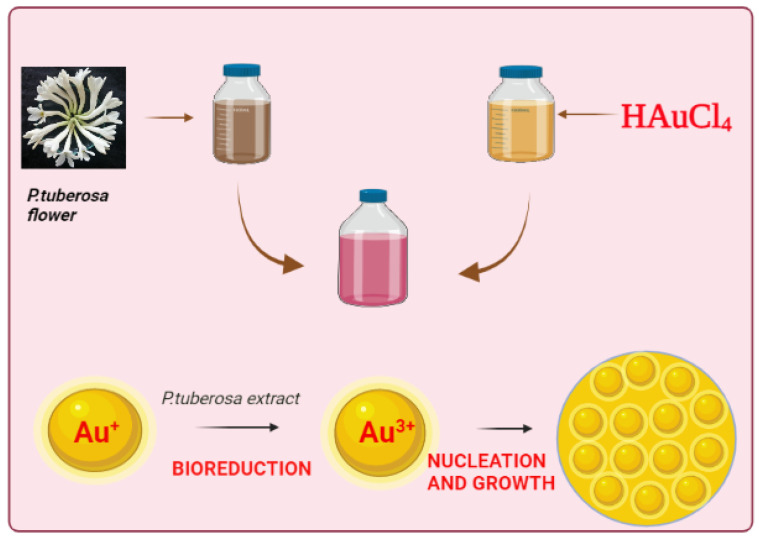
A schematic illustration of the PtubAuNPs.

To synthesize the PtubAuNPs, we preliminarily optimized the various factors, and finally the parameters were fixed as: flower extract of 3 mL; HAuCl_4_ of 1.5 mM; temperature of 60 °C; pH 9; time of 60 min; and stoichiometric proportion (7:3) for fine generation of the PtubAuNPs. As the reaction proceeded, a gradual change in the color was visually observed at the time intervals. After 60 min, a deep purple-red color formation was observed, which indicated the synthesis of the PtubAuNPs. The color change was due to the reduction of HAuCl_4_ into nanoparticles due to the presence of phytochemicals present in the flower extracts [39] (Figure 2a). The chloroauric acid measured SPR at 200–250 nm, due to the presence of raw materials (nitric acid and HCL). Further, a UV–vis spectroscopy analysis was performed. The recorded spectrum displayed an intense strong band at 543 nm (Figure 2b); this was due to the phenomenon of surface plasmon resonance (SPR). This is peculiar to metallic nanoparticles, which produce strong electromagnetic fields on the surface of the particles, which in turn reflect as scattering and absorption [40]. Finally, at the optimized parameters, the reaction initiated slowly, followed by growth and nucleation in the synthesis of the PtubAuNPs (Figure 3). 

**Figure 4:**
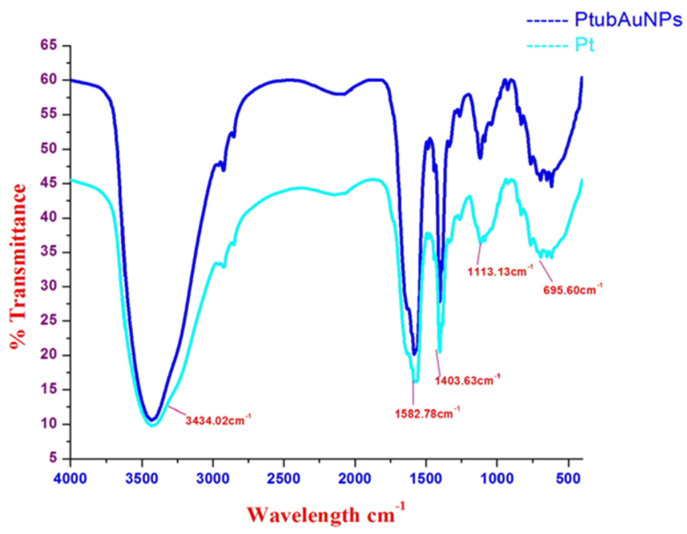
The FTIR spectra of the plant extract and AuNPs.

FTIR is an analytical technique that identified the responsible functional groups involved in the reduction and synthesizing of the gold nanoparticles. The FTIR spectrum is shown in Figure 4. The IR spectra of the plant extracts and PtubAuNPs are represented. The band observed at 3434 cm^−1^ corresponded to the –OH groups and the sharp band 1582 cm^−1^ represented the presence of the amide group. The structural changes at 1582 cm^−1^ were due to the coordination of the N-amide group with gold ions. A sharp hinge at 1403 cm^−1^ was related to the presence of C=C aromatic rings, which might be attributed to the presence of phenolic compounds. The distinct peak at 1113 cm^−1^ showed the presence of the C-O hydroxyl ester group, and the weak vibration at 695 cm^−1^ was confined to the alkyl halides group. Overall, based on the comparison of the spectra of PtubAuNPs and Pt extract with the support of the literature, it was plausibly confirmed that synthesis of gold nanoparticles was obtained due to the presence of bioactive metabolites and proteins that contained amines, phenol, alcohol, ester linkages, and carboxylic acid functional groups [41].

**Figure 5:**
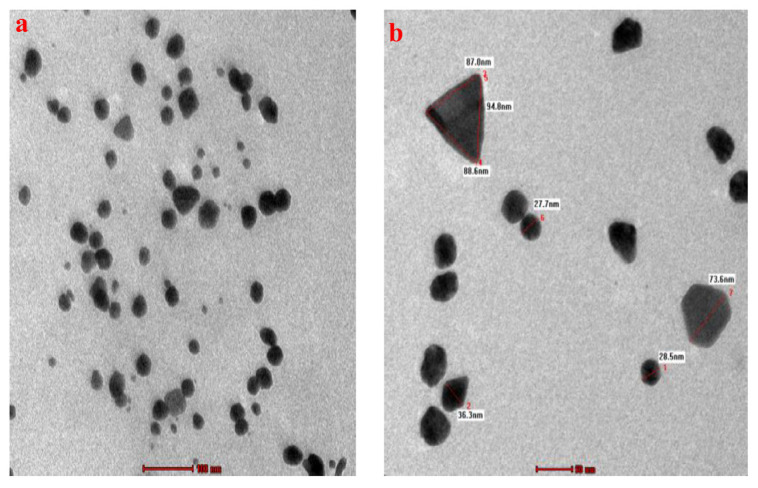
(**a**,**b**) TEM images of the PtubAuNPs.

**Figure 6:**
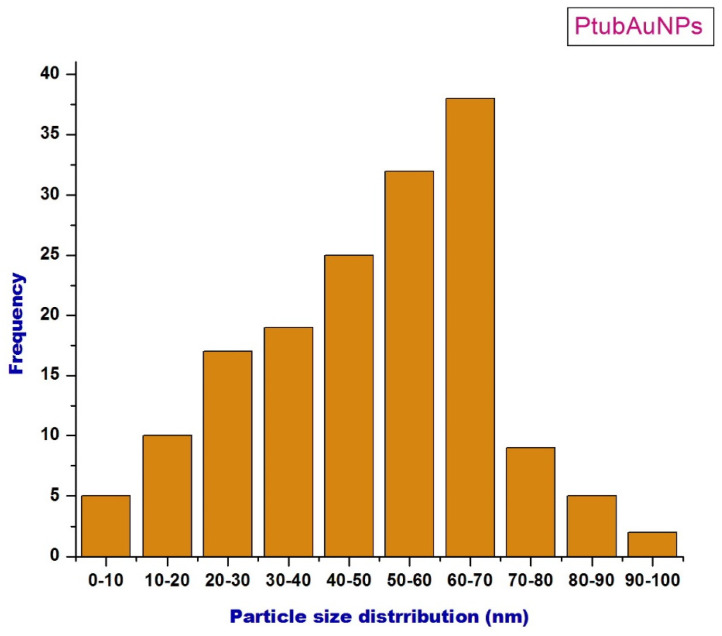
The particle size histogram of the PtubAuNPs analyzed using the Image J software.

**Figure 7:**
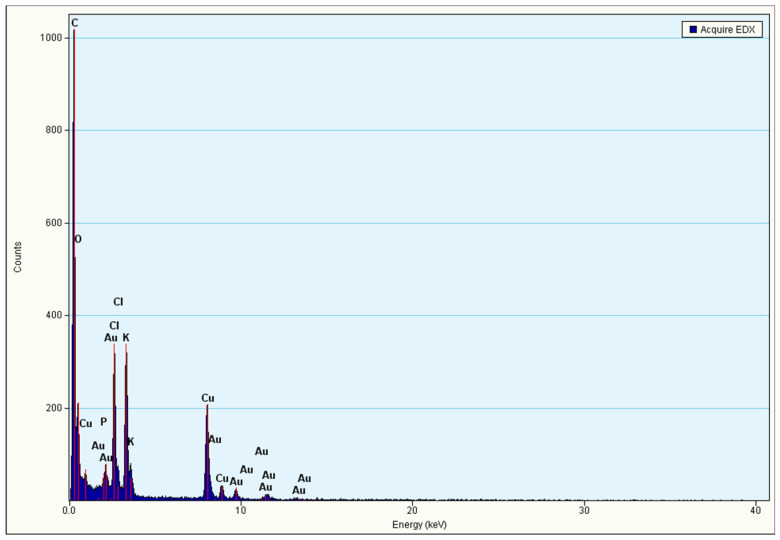
The EDX analysis of the PtubAuNPs.

The morphology (size and shape) and the associated particle size histogram of the PtubAuNPs were determined by transmission electron microscopy analysis. In Figure 5, the images depict the surface morphology of the PtubAuNPs. The synthesized PtubAuNPs were exhibited in various forms, such as spheres, triangles, pentagons, hexagons, and rods. A similar observation was made of the *Lonicera japonica* flower-mediated gold [34]. The production of various geometric shapes of the nanoparticles was primarily due to the chemical constituents present in the extract. Botteon et al. (2021) [42] claimed that nanoparticles with different geometric sizes were larger than spherical-shaped nanoparticles due to the low quantum of biomolecules responsible for capping and stabilization, which led to the formation of large anisotropic nanoparticles. The particle size histogram, which portrays particles of varying size with an average size of 38.76 nm (Figure 6), was generated by using the Image J software. The elemental composition of the PtubAuNPs was analyzed by using EDX; from the spectrum, an absorption band that was characteristic of gold was shown; while the other weaker bands for chlorine, potassium, and chloride were traced from biomolecules of *P. tuberosa* and chloroauric acid (Figure 7).

**Figure 8:**
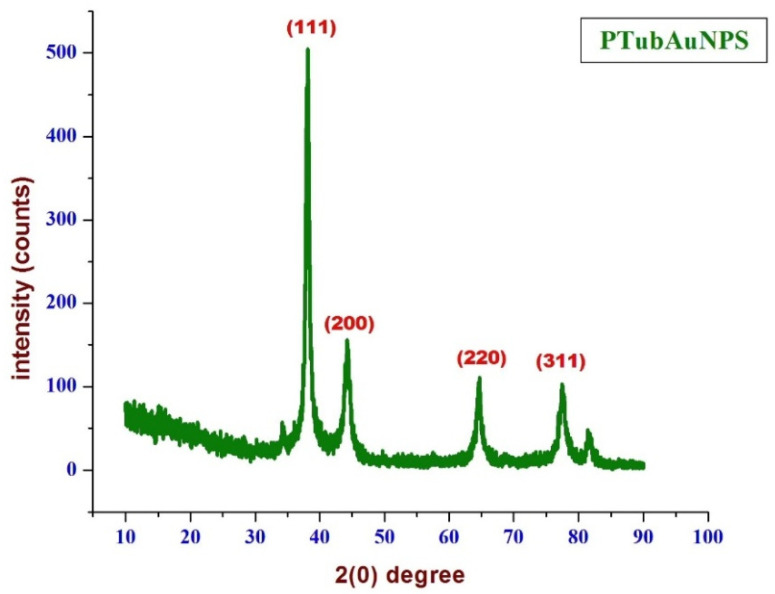
The XRD spectrum of the PtubAuNPs.

The XRD diffraction spectrum (Figure 8) that corresponded to the peaks at 38.05°, 44.28°, 64.43°, 77.37°, and 81.55° could be indexed to the planes of (111), (200), (220) and (311), respectively, representing the face crystalline nature (fcc) of the PtubAuNPs according to the Joint Committee on Powder Diffraction Standards (JCPDS; file no. ICDD-PDF2, Release 2007, PA, USA, 2007).

**Figure 9:**
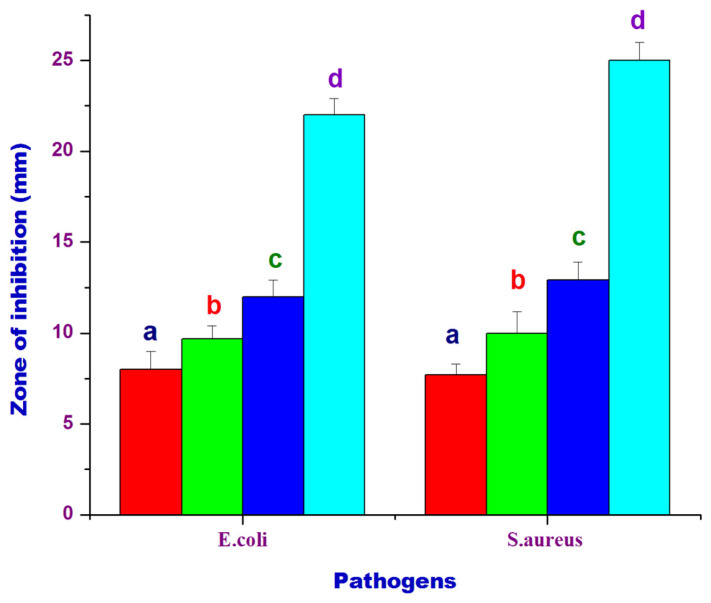
Antimicrobial activity of PtubAuNPs: (a) 25 µg/mL; (b) 50 µg/mL; (c) 75 µg/mL. The control (d) was 20 µg/mL of gentamicin. Means ± SE followed by different letters (a–d) within the same row were significantly different (Tukey’s test, *p* < 0.05).

The antibacterial activity of the PtubAuNPs is shown in Figure 9. In the figure, it is clearly evident that the PtubAuNPs exerted an antibacterial effect in a dose-dependent manner. The zone of inhibition (ZOI) was measured for *Escherichia coli* (10 mm > 11 mm > 13 mm) and *Staphylococcus aureus* (7.7 mm > 10 mm > 12.9 mm) with respect to the concentrations (25 µg > 50 µg > 75 µg). Significant variations of ZOI were observed between *E. coli* and *S. aureus*. This was due to the difference in structural architecture; Gram-positive bacteria (*S. aureus*) possess a thick peptidoglycan layer, whereas Gram-negative bacteria (*E. coli*) have a thin layer membrane; which acts as a barrier for penetration of nanoparticles [43]. Gold nanoparticles inherently possess excellent antibacterial activity. The mechanistic action of gold nanoparticles in bacterial cells is via the following pathways: (i) the gold nanoparticles adsorb to the negatively charged cell membrane and cause generation of reactive oxygen species (ROS), which in turn leads to damage to cellular respiration chain, damage to DNA/proteins and cellular synthetase activity, and finally cell death [44]. The use of gold nanoparticles is exponentially increasing in treating bacterial infections due to the particles’ size and ability to overcome bacterial resistance. Lu et al. (2017) developed AuNPs conjugated with vancomycin and evaluated them in *E. coli* and *S. aureus*; the AuNPs–vancomycin conjugate showed superior antibacterial activity compared to bare vancomycin [45].

**Figure 10:**
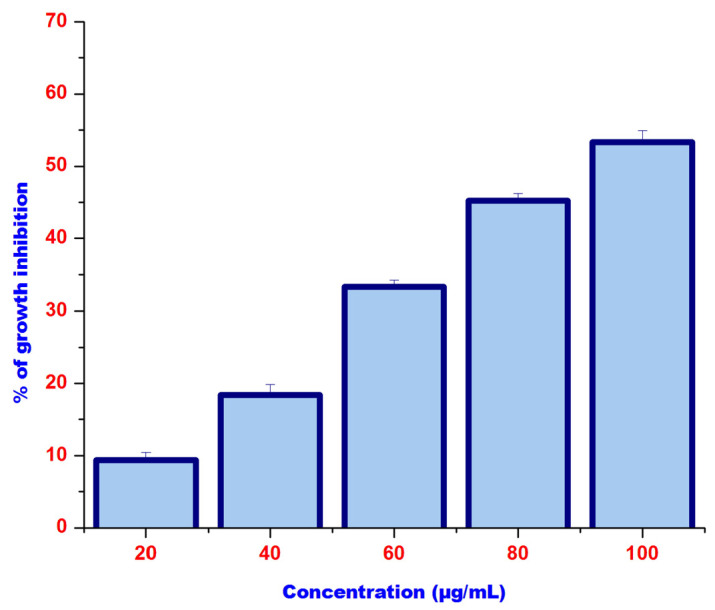
The MTT assay of the PtubAuNPs at concentrations of 0–100 µg/mL. The results are expressed as mean ± SD of the three replicates.

The efficacy of PtubAuNPs in triggering the toxicity in the MCF 7 cell line was assessed by MTT assay. This was a basic preliminary tool to understand the cytotoxic action of AuNPs against MCF 7 cell line. As per the MTT assay, the PtubAuNPs progressively created toxic effects with respect to the dose concentration method, as shown in Figure 10. After 24 h of treatment, the MCF 7 cell growth was inhibited by the PtubAuNPs with the LC_50_ 100 µg/mL. The anticancer effect was due to the molecules present in the extract that coated the surfaces of the AuNPs. The speculated mechanistic activity of the gold nanoparticles was by induction of ROS followed by up- and downregulation of proapoptotic proteins (Bid, Bax) and antiapoptotic proteins (Bcl 2, Bcl-xl), leading to dysfunction of the mitochondrial membrane potential, and finally cell death [46]. The above model of the apoptotic event was observed in *Curcuma wenyujin* extract-mediated gold nanoparticles in human renal cell carcinoma A498 cells. Henceforth, PtubAuNPs exerted their anticancer activity by the generation of ROS and apoptosis [47].

## 4. Conclusions

The present study was a preliminary study to determine the outcome for potential biomedical applications of PtubAuNPs. The PtubAuNPs were synthesized in a single one-step method and exclusively characterized with UV–vis spectroscopy, FTIR, TEM, and XRD, which portrayed their physico-chemical properties. The antimicrobial activity of PtubAuNPs against *E. coli* and *S. aureus* was splendid. The cytotoxic response of the PtubAuNPs against MCF 7 was remarkable and significant. Based on the above scientific outputs, the PtubAuNPs can further proceed in detailed investigations at the molecular level and animal models to calibrate the PtubAuNPs as a safe therapeutic agent in infectious/noninfectious diseases.

## Figures and Tables

**Table 1:** The gold nanoparticles synthesized using flowers.

Flower	Shape and Size	References
*Cassia auriculata*	Spherical, hexagonal, triangular; 10–55 nm	[19]
*Achillea wilhelmsii*	Spherical; 70 nm	[20]
*Gnidia glauca*	Spherical; 50–150 nm	[21]
*Nyctanthes arbortristis*	Spherical; 20 nm	[22]
*Plumeria alba*	Spherical; 20–30 nm	[23]
*Tussilago farfara*	Spherical; 19 nm	[24]
*Mangifera indica*	Spherical; 10–60 nm	[25]
*Hibiscus sabdariffa*	Spherical; 15–45 nm.	[26]
*Caesalpinia pulcherrima* (peacock)	Spherical; 10–50 nm	[27]
*Couroupita guianensis*	Spherical, triangular, tetragonal, and pentagonal with irregular contours; 7–48 nm.	[28]
*Mimosa pudica*	Spherical; 25 nm	[29]
*Mirabilis jalapa*	Spherical; 114 nm	[30]
*Musa acuminata colla*	Spherical; 10–15 nm	[31]
*Peltophorum pterocarpum*	Spherical; 30–50 nm	[32]
*Carthamus tinctorius* L. (Saf) *s*	Spherical, triangle; 40–200 nm	[33]
*Lonicera japonica*	Spherical, triangle, hexagonal; 50–60 nm	[34]
*echinacea*	Spherical; 80–120 nm	[35]
*Anthemis xylopoda s*	Spherical	[36]
*Helianthus annuus*	Spherical; 30–50 nm	[37]
*Rosa* sp. *petals*	Spherical; 3–15 nm	[38]
